# Cloud-Based Lung Tumor Detection and Stage Classification Using Deep Learning Techniques

**DOI:** 10.1155/2022/4185835

**Published:** 2022-01-10

**Authors:** Gopi Kasinathan, Selvakumar Jayakumar

**Affiliations:** Dept. of ECE, SRMIST, Chennai, India

## Abstract

Artificial intelligence (AI), Internet of Things (IoT), and the cloud computing have recently become widely used in the healthcare sector, which aid in better decision-making for a radiologist. PET imaging or positron emission tomography is one of the most reliable approaches for a radiologist to diagnosing many cancers, including lung tumor. In this work, we proposed stage classification of lung tumor which is a more challenging task in computer-aided diagnosis. As a result, a modified computer-aided diagnosis is being considered as a way to reduce the heavy workloads and second opinion to radiologists. In this paper, we present a strategy for classifying and validating different stages of lung tumor progression, as well as a deep neural model and data collection using cloud system for categorizing phases of pulmonary illness. The proposed system presents a Cloud-based Lung Tumor Detector and Stage Classifier (Cloud-LTDSC) as a hybrid technique for PET/CT images. The proposed Cloud-LTDSC initially developed the active contour model as lung tumor segmentation, and multilayer convolutional neural network (M-CNN) for classifying different stages of lung cancer has been modelled and validated with standard benchmark images. The performance of the presented technique is evaluated using a benchmark image LIDC-IDRI dataset of 50 low doses and also utilized the lung CT DICOM images. Compared with existing techniques in the literature, our proposed method achieved good result for the performance metrics accuracy, recall, and precision evaluated. Under numerous aspects, our proposed approach produces superior outcomes on all of the applied dataset images. Furthermore, the experimental result achieves an average lung tumor stage classification accuracy of 97%-99.1% and an average of 98.6% which is significantly higher than the other existing techniques.

## 1. Introduction

Recently, the COVID-19 pandemic affected many people around the world. The current scenario transformed the entire healthcare industry into electronic diagnosis, remote access, virtual consultant, and E-treatment to avoid physical personalization and to reduce the disease spread. This pandemic has caused a new level of severity in the healthcare industry, increasing the death rate of chronic disease patients, particularly those affected with cancer, diabetes, and cardiac diseases, due to a shortage of physicians, caregivers, and radiologists [1]. The death rate from cancer and other chronic diseases is rising every year around the world. According to the WHO [2], lung cancer patients had the highest death rate, followed by breast cancer patients. Lung tumor remains the leading cause of tumor death rate, with the estimation of 1.80 million deaths (18%), and the global tumor burden was expected as around 28.40 million cases in 2040 and also rises 48% in 2020, which was a huge increase in transitioning (from 65% to 94%) versus transitioned (33% to 57%) countries due to the cause of demographic change, although this will be further exacerbated by increasing in risk factor which is associated with globalization and for an economical growth [3]. There are standard challenges on recognizing the lung tumors on patients from a decade like zero symptoms irrelevant to age factor, patients suffering from breathing problem, and patients affected with 30-40 years of smoking, which are very critical to identify in their early stages [4, 5]. In addition, inconsistent treatment and monitoring raise the risk of death. Many researchers have addressed these challenges on detection of lung cancer through various techniques called segmentation, detection, and classification approaches [6–8]. Artificial intelligence plays a significant role in computer vision, big data, and healthcare applications in recent days due to its high-level performance in detection and prediction and is most suitable for classification problems. Chaturvedi et al. adopted the AI-based supervised learning models for lung tumor identification and classified them as malignant or benign [9]. Valluru and Jeya developed multilayer preceptor, SVM, and KNN classifiers as binary lung tumor classifier [10]. Serj et al. proposed a grey wolf optimization algorithm with the combination of genetic algorithm as hybrid lung tumor classifier [11]. But still, there are few limitations of the conventional approaches that are not sufficient in early detection, less efficient in accuracy rate, not suitable for stage classification, etc.

**Figure 1:**
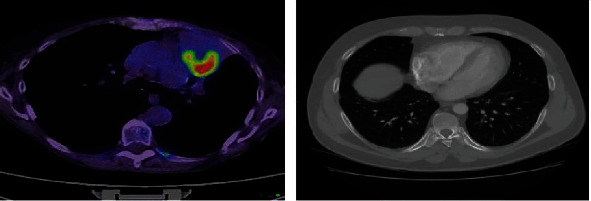
(a) F-FDG PET image; (b) LIDC-IDRI DICOM lung image.

To address these challenges researchers have initiated to develop depth networks like convolutional neural network (CNN) and reinforcement learning model (RNN) as unsupervised lung tumor detectors, predictors, and classification modules. Serj et al. developed CNN as a binary classifier for the KDSB17 dataset in detection of lung cancer. The DCNN structure is a 4-layer architecture with ReLU activation function [11]. Liu et al. proposed a reinforcement Q-learning algorithm for tumor detection and classified its stages as malignant or benign. The authors addressed the challenges on designing the RNN model for lung tumor detection in terms of localization [12]. Lusfabricio et al. [13] created a mask RNN model that goes through the lung segmentation process to build a respiratory map and then uses fine-tuning to locate the border of pulmonary nodules on the DICOM CT lung image. The limitations of the existing lung cancer detection methods are inefficient for analyzing the large-scale database which leads to high performance in accuracy rate, F1-score, sensitivity, recall, and precision metrics. The major drawbacks of these models are unable to fit in the current pandemic scenario where the physical medication is impossible. The advancements on Internet of Things, cloud server system, big-data techniques, and sensor units are able to improve the electronic diagnosis, prediction of lung cancer on early stage, detection of localization, tumor segmentation, stages classification, regular monitoring, E-treatment, etc. We are motivated by these advanced technique performance and adopted them to utilize in the healthcare industry. In this work, we addressed these current scenario challenges and designed a new prototype called “Cloud-based Lung Tumor Detector and Stage Classifier” (Cloud-LTDSC) framework which detects lung cancer as early as possible based on the large-scale database which comprises of both PET scan data (Figure 1(a)) and LIDC-IDRI DICOM images (Figure 1(b)).

### 1.1. Significant Contribution

To detect non-small-cell lung cancer (NSCLC) and to reduce the overall death rate, we proposed an intellectual diagnosis module called Cloud-LTDSC. The proposed lung tumor detector used unsupervised learning approaches for segmentation, feature extraction, and stage classification. Also, we proposed a new cloud system for database storage, processing the proposed modules, and remote monitoring with E-treatment, which reduces time complexity, improve detection rate, and provides regular diagnosis with medications.

- Initially, the LIDC-CT DICOM images and PET scans are analyzed in terms of noise and memory requirement, which is detailed in our own previous work [14]
- The proposed system is a hybrid model which is introduced for effective analysis, and this model dependency is in between clinical feature and image-quantitative feature, and also, it generates a new feature representation for more accurate computer-aided diagnosis
- This method uses multilayer convolutional neural network (M-CNN) for an effective tumor stage classification
- In this paper, we conducted various numbers of experiments with the help of several medical datasets and also use real-time data which was collected directly from the patients through a cloud device
- The proposed Cloud-LTDSC has been conducted for performance measures of a new method which also exists in this paper

The paper is structured as follows: relevant literatures are reviewed and discussed in Section 2; methodologies are detailed in Section 3. The methodology of this approach was explained and contextualized. This section is classified into (1) dataset (Section 3.1), (2) lung tumor segmentation using ACM (Section 3.2), (3) cross-validation set (Section 3.3), and (4) lung tumor classification using M-CNN which is elaborated in Section 3.4, and LC- Cloud framework is described in Section 3.5, and performance metrics are illustrated in Section 5.

## 2. Related Works

For lung cancer diagnosis, Joshua et al. introduced the 3D CNN unsupervised learning model [15]. 3D CNN is a binary classifier model with an enhanced gradient activation function that improves lung tumor visibility. The proposed AlexNet detection algorithm is compared to an existing 2D CNN learning classifier and tested using the LUNA dataset. The suggested model is ineffective due to a paucity of testing data, with only 10% of the training database being used.

Chaunzwa et al. created a supervised CNN predictor for detecting early-stage adenocarcinoma (ADC) and squamous cell carcinoma (SCC) in lung cancer patients. CNN has been validated using real-time non-SCLC patient data acquired at Massachusetts General Hospital from early-stage affected patients [16]. There are 311 data phases in the database that have been collected. The constructed CNN, which is a VGG network-based learning predictor, had a prediction rate of 71 percent AUC, which was insufficient. The VGG CNN model's shortcoming is that it has not been preprocessed in terms of noise removal and CT image segmentation, which surges the prediction rate.

Chaturvedi et al. assessed the latest lung cancer detection and categorization strategies. The latest lung nodule diagnosis, localization, and classifiers with standard datasets LIDC-IDRI, LUNA 16, and Super Bowl Dataset 2016 are familiar with supervised learning algorithms such as SVM, KNN, and CNN. These are the most frequent and typical threshold CT data used for disease diagnosis, according to the authors in [9]. Kalaivani et al. introduced the DenseNet model, a binary classifier based on a deep CNN network for detecting malignant or benign lung cancer patients [17]. The researchers employed a dataset of 201 lung scans, with 85 percent of the photos being used for training and 15 percent being used for testing and classification. The proposed method obtained an accuracy of 90.85% in tests, according to the results.

The authors evaluated histopathological pictures; a classification system was developed to distinguish between 5 types of lung and colon tissues (2 benign and 3 malignant). The obtained results suggest that the proposed model can accurately detect cancer tissues up to 96.33 percent of the time [18]. Qin et al. described how to evaluate EGFR mutation status via computer-assisted diagnostics, which includes extracting, analyzing, and fusing multitype interdependent features. A new hybrid network model built of CNN-RNN architecture is involved in this study. Image quantitative characteristics are extracted using CNN, and the relationship between different types of features is modelled [19].

When compared to the conventional features extracted, their findings showed that multitype dependency-based feature representations outperform single-type feature representations (accuracy = 75%, AUC = 0.78). For supporting radiologists, Masood et al. developed an approach using computer-aided diagnosis support systems for lung nodule diagnosis based on 3D-DCNN. The LUNA16, ANODE09, and LIDC-IDRI datasets were used to train and validate a computer-aided diagnosis (CAD) system in this study [20]. To categorize distinct forms of cancer based on tumor RNA sequences of genomic data, ElNabi et al. offer a unique optimised deep learning strategy based on binary particle swarm optimization with decision tree (BPSO-DT) and convolutional neural network (CNN). The performance criteria such as recall, precision, and F1-score were mentioned in this study [21]. Label-free approaches, according to Abdulgani and Al Ahmad, do not induce cell harm or lead to any changes in cell composition or inherent traits. This research used advances in optical measurements with Prony approaches to improve cell classification using measured optical profiles [22]. By the improvement of Tobacco Exposure Pattern (TEP) classification models and uncovering their interaction linkages at multiple biological levels, He et al. were able to identify signature genes. TTZ is a new method for extracting existing features as an input variable to the TEP classification model [23]. To train and evaluate the TEP classification model, with two independent LUAD datasets, 34 genes were recognised as tobacco-related mutation signature gene, with an accuracy of 94.65 percent for training data and 91.85 percent for validation data.  Table 1 represents the summary of the recent models and dataset used for performing various algorithms.

## 3. Proposed Methodology

**Figure 2:**
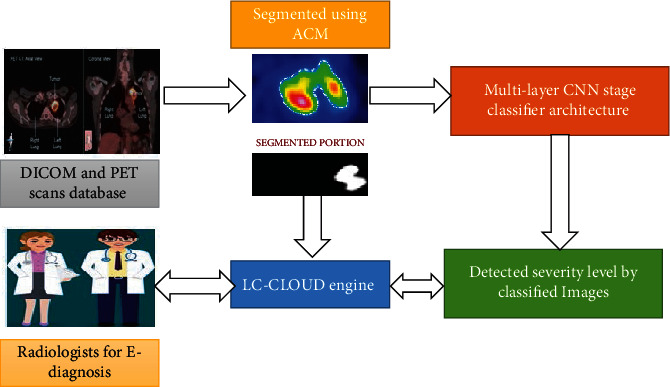
Overview of the proposed Cloud-LTDSC framework.

To achieve accurate result in terms of “accuracy, precision, and recall,” the recommended Cloud-LTDSC hybrid framework includes lung tumor detector, lung tumor segmentation, and stage classifier modules.  Figure 2 shows a summary of the suggested model, followed by descriptions.

### 3.1. Dataset

#### 3.1.1. Image Acquisition

F-FDG PET/CT scans of 94 individuals with NSCLC were used in this investigation. Between March 2010 and April 2014, the images were taken. Siemens Biograph 6, HiRez, was the PET/CT imaging device used. F-FDG was administered into the patients in doses of 10 to 15 mCi, and PET/CT scans took about 60 minutes. PET scan was done for every 2 to 3 minutes of the 8 or 9 bed positions. The photos were reconstructed using a three-dimensional iterative reconstruction method. Using eSoft software (eSS) platform, there are two experts in the field of nuclear medicine that reviewed the photos and for an entire body projection (Siemens, USA). The Research Ethic Committee of KRTH accepted this work. All study participants were given the opportunity to give their informed consent. There were seven female patients and 92 male patients (average age was 59; interval of age is between 36 and 82). There has been no research on tumor variability in male and female NSCLC patients until now. As a result, we did not examine the effect of gender on cancer subtype characteristics when designing this study. Forty-five patients were found to have an ADC, while the others were found to have SqCC. The Lung Image Database Consortium (LIDC-IDRI) data collection provided the DICOM CT lung images [24]. Each DICOM lung CT scan is saved in the DICOM file formats, which is having 512 × 512 pixels in size. Images of tissue slices with a thickness of 1.15 mm to 2.75 mm and a diameter of 0.45 mm to 0.75 mm are included in this dataset [25]. Each radiologist saw all CT scans individually and labelled lesion that fit into one of three categories nodules, seminodules, and nonnodules. Four radiologists looked at the CT scans and identified the tumor types. This LIDC/IDRI dataset included 1024 cases, 358 CT scans of lung nodules, and an XML file with the results of a 2phase of image-annotation processes by a radiologist. A total of 623 pictures of lung nodule-free and nodule-filled slices were used. In this model, nodules with diameters ranging from 4 to 20 mm (125 pixels∗125 pixels) were used. The LIDC-IDRI data collection was used to create all DICOM CT lung images with solid nodules, semisolid nodules, and nonsolid nodules referred by few radiologists.

Selected nodules were classified as “well-circumscribed, juxtavascular, juxtapleural, pleural tail.” The ACM approach centred a region of interest (lung tumor) with a nodule size of 75 × 85 × 45 mm. As described by a few radiologists, the size of the nodule may be retrieved, and the number of voxels that engaged all dimensions can be determined. Then, they pointed to the nodule's centre, and 55 squares were suggested as the zero-level set using the Signed Distance level set Function (SDF), which aids in the conversion of lung images into 3D shapes. The real and simulated nodule characteristics are listed in Table 2. The total number of images with respect to solid nodules, semisolid nodules, and nonsolid nodules is given in Table 3.

### 3.2. Lung Tumor Segmentation Using Active Contour Model

In comparison to other organs such as the brain, detecting lung nodule in a DICOM CT lung imaging is tough. Likely, bronchus and intensity of lung nodule identification in a CT DICOM lung image are tedious. The bronchus and intensities of blood vessels within the tumor area are then measured, and the region of lung parenchyma is partitioned from back to front. The steps involved for the detection of lung tumor are as follows:

1. Remove the region of mediastinum and thoracic wall if the lung parenchyma was not reconstructed
2. The tumor part of the lung picture is segmented using the active contour model (ACM)
3. A total of 925 lesions from different patients were collected, together with nodule information

The number of different forms of benign nodules is shown in Table 2. Due to a gradient value, the edge-based ACM causes some segmentation modifications. The leaking problem occurs when there is a weak boundary in the image [26]. The approach of edge-based segmentation is not possible due to noise in the image border. According to Rouhi et al., border leakage does not occur in region-based ACM, and undesired sections of the picture are removed. When the roundness rule was applied to the objects, the result was approximately 1, resulting in a solid nodule [27].

Snake's model was utilized to develop a curve that aids in the detection of a tumor portion in the relevant photos. The curve must be drawn around the identified item, and then, it must alter its location towards the interior and end at the object borders [28]. For each contour line that gathers a similar amount of control point and distinct point, the initial prediction model of active contour is utilized; it predicts contour point that helps for the contour lines and then generates the active contour model's first prediction [29]. The proposed approach generates 3D features from 2D stochastic characteristics, which are then fed into the CNN classifier.

Considering gray-scale DICOM image [(*y*, *z*)€ *R*2], the contour of the segmented portion was mentioned as

(1) $$\begin{array}{c} C_{k}\left(s\right)={\left[Y_{k}\left(s\right),Z_{K}\left(s\right)\right]}^{T},\left\{s\in \left(0,1\right)\right\}. \end{array}$$

In the proposed ACM, we reduced the number of points to fit the curve in the tumor portion which is segmented. Let the sample contour *C*_*k*_(*s*) be into the given *n* points, and the overall curve *U*_*k*_ was given by

(2) $$\begin{array}{c} U_{k}=\left\{C_{k,1},C_{k,2},\cdots \cdots ..C_{k,n}\right\}. \end{array}$$

The modified ACM is evaluated using a gradient value which helps for an edge detection. It is derived from the image as

(3) $$\begin{array}{c} E_{k}\left(C\right)=\propto \int_{0}^{1}{\left|C^{\prime }\left(s\right)\right|}^{2}ds+\beta \;\int_{0}^{1}\;\left|C^{\prime \prime }\left(s\right)\right|ds-\mu \int_{0}^{1}{\left|\nabla f_{0}\left(C\left(y\right)\right)\right|}^{2}dy, \end{array}$$

*α*, *β*, and *μ* are constant, and *E*_*k*_ refers to the parametric curve with the *k*th image.

Using the Mumford-Shah model, we derived an expression for intensity outside and inside curves with level set method and energy approximation *A*_E_ is given by,

(4) $$\begin{array}{c} A_{\text{E}}\left(I_{1},I_{2},\emptyset \right)=\int_{\Omega }{\left(f\left(y\right)-I_{1}\right)}^{2}J_{a}\left(\emptyset \right)dy+\int_{\Omega }{\left(f\left(y\right)-I_{2}\right)}^{2}J_{a}\left(-\emptyset \right)dy+\gamma \int_{\Omega }J_{a}\left(\emptyset \right)dy+\beta \int_{\Omega }\delta \left(\emptyset \right)\left|\nabla \left(\emptyset \right)\right|dy. \end{array}$$

Using the Euler-Lagrange equation, inside and outside curve intensities *I*_1_ and *I*_2_ are expressed as

(5) $$\begin{array}{c} I_{1}\left(\emptyset \right)=\frac{\int_{\Omega }f\left(y\right)*J_{a}\left(\emptyset \left(y\right)\right)dy}{\int_{\Omega }J_{a}\left(\emptyset \left(y\right)\right)dy}, \end{array}$$

(6) $$\begin{array}{c} I_{2}\left(\emptyset \right)=\frac{\int_{\Omega }f\left(y\right)*\left(1-J_{a}\left(\emptyset \left(y\right)\right)\right)dy}{\int_{\Omega }\left(1-J_{a}\left(\emptyset \left(y\right)\right)\right)dy}. \end{array}$$

**Figure 3:**
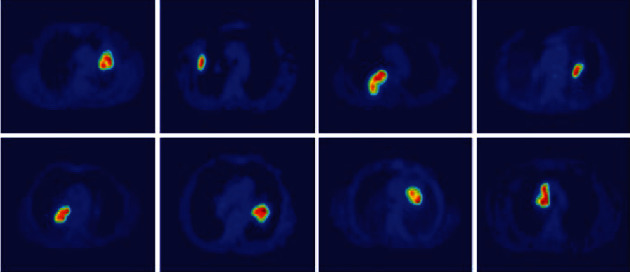
Segmentation of PET images.

**Figure 4:**
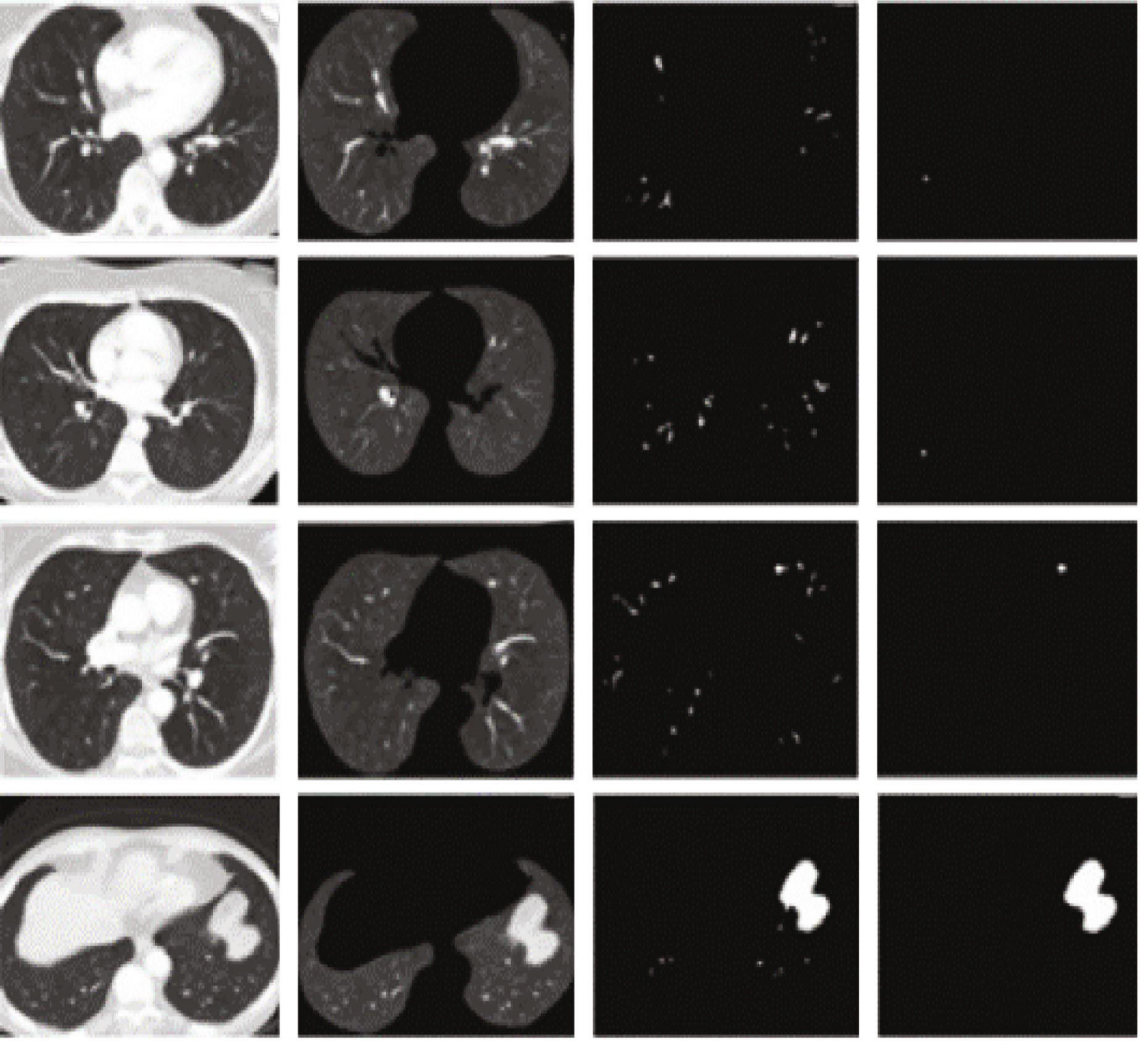
Segmentation of the LIDC-IDRI dataset.

These expressions (5) and (6) give the weight calculation which is given for inside and outside curve intensities *I*_1_ and *I*_2_ which is derived by decreased kernel function *f*(*y*). It becomes constant and represented average intensities to be determined.  Figure 3 shows the resultant segmentation image of PET scan. Using the LIDC-IDRI dataset, we segmented the tumor portion which is shown in Figure 4.

### 3.3. Cross-Validation Set

**Figure 5:**
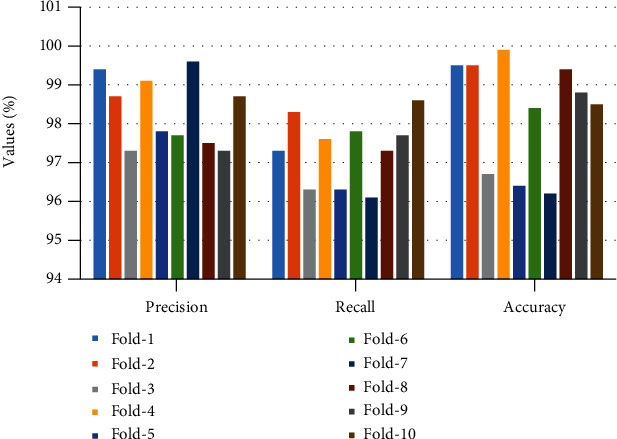
Percentage of precision, recall, and accuracy for 10-fold cross-validation.

**Figure 6:**
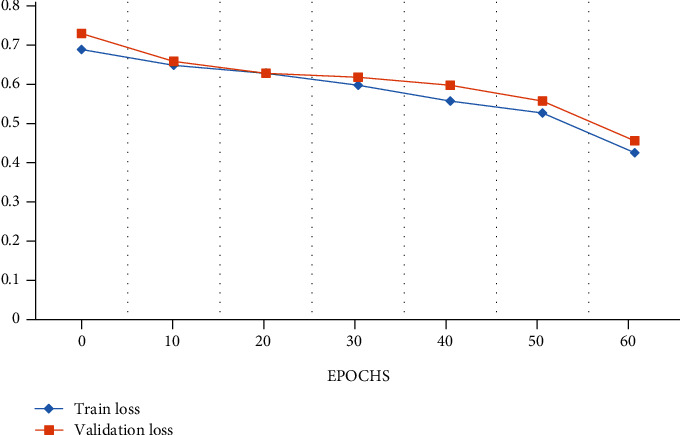
Performance analysis graph for loss with epochs.

**Figure 7:**
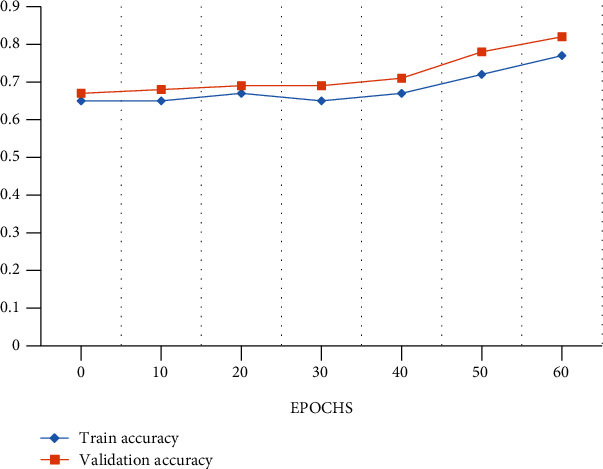
Performance analysis graph for accuracy with epochs.

In this paper, 10-fold cross-validation method is widely utilized for the LIDC-IDRI/PET dataset with performance metrics which is shown in Figure 5. The dataset was erratically splitting in this work into stratified 10-fold cross-validation, which has been employed in many deep convolutional neural networks and traditional machine learning-based bioimage and biosignal studies. M-CNN model was trained from scratches and also starts with some random weight using a processor NVIDIA GeForce GTX 1650 PC for a High-Performance Computing (HPC). The LIDC/IDRI and PET scan images such as 1018 DICOM CT scans and 355 PET scans were grouped as 10-fold cross-validation sets. In this set, 9 sets are for training and 1 set is for validation with a repeat of 15 epochs. The number of epoch for overall training processes will be 150 with a batch size of 10 from the given dataset, and early stopping approaches and also regularization were used to prevent from an overfitting. In the cross-validation set, we fold our dataset into 10-fold set of images. In fold 3, fold 5, and fold 7, we have used PET scan images for testing; we got less accuracy than other fold sets as we achieved. From Figure 6, the performance analysis of training data loss and validation data loss with respect to 60 epochs for 4-fold validation set is clearly shown. And also, we clearly show the accuracy analysis with respect to 60 epochs for training and for 4-fold validation data in Figure 7.  Table 4 shows the performance metrics of accuracy, precision, and recall for 10-fold cross-validation with the mean value.

### 3.4. Lung Tumor Stage Classification

Artificial neural networks are stimulated by the human brain which meets machine learning for solving complex problems. So, machine learning has a subset called deep learning (DL).

**Figure 8:**
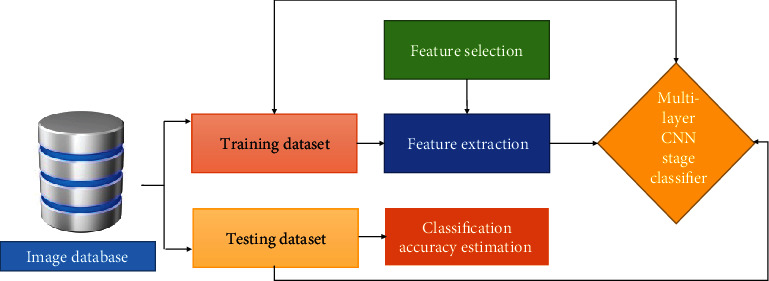
Classification based on image database.

DL method is used to draw out features from bulk data; collecting useful information from big data using DL algorithms is useful in many aspects. Since detecting a feature is time-consuming and costs a lot, DL methods do not require labelled data for learning objectives. In this case, we may have both types of data labelled and unlabelled in healthcare like CT Scan images regardless of a medical condition, huge data that is not labelled, etc. The classification based on image database is shown in Figure 8.

**Figure 9:**
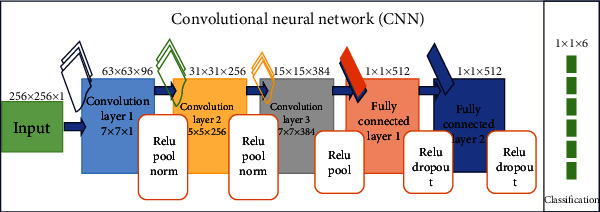
CNN architecture.

There are many deep learning techniques. In this portion, we discussed some of the widespread techniques among them. (1) Artificial neural network (ANN) is a DL technique that contains several organized layers which have perceptron, the neurons. The convolutional layer is one of the most important components of CNN. Convolution is a mathematical procedure for combining two sets of data. Convolution is connected to an input data using convolution filters to create a feature map. Convolution is accomplished by sliding this filter over the input. We conduct element-wise matrix multiplication and total the results at each position. Pooling is frequently done after a convolution process to minimise dimensionality. This allows us to limit the number of parameters, reducing training time and preventing overfitting. Each feature map is downsampled independently using a pooling layer, lowering the height and breadth while maintaining the depth. Dropout is only applicable to input and hidden layer nodes. The edges to and from the nodes have been thrown. The use of multilayer network is for the hidden layer in which neurons are not directly linked to the output layer and also used in multilayer network to address the classification issue for a nonlinear data. The hidden layers will be understood geometrically as an extra hyperplane that increases the network layer separation capability. Multilayer networks were once utilized in computer vision, but convolutional neural networks have now taken their place. This network is no longer considered as adequate for sophisticated computer vision applications. Each perceptron was connected to any other perceptron, giving it the property of completely connected layer. The total number of parameters might get quite large which are all the disadvantages. Even though there is duplication in such large dimensions, this is ineffective. It also ignores geographical information, which is a drawback. Its inputs are flattened vector. Multiple layers are starting from the input layer then the hidden layer; actually, this works as the training layer and output layer. If hidden layers are increased vastly, then it may not assure improved results. Overfitting also takes place if many layers are added at the same time; as a result, many distortions forward too much interference in data being captured. Convolutional neural network (CNN) until now highlighted beneficiary technique in healthcare (Figure 9) [27]. A static extent flight path is using as an input. It can be applied to medical data like in image processing for the detection of the lung tumor. Perceptron interconnected with each other and assigned a weight to them that can be adjusted after each iteration. As the propagating waves are transmitted in one direction, i.e., starting from input meanwhile ending on the output layer, this is known as a feed-forward network.

**Figure 10:**
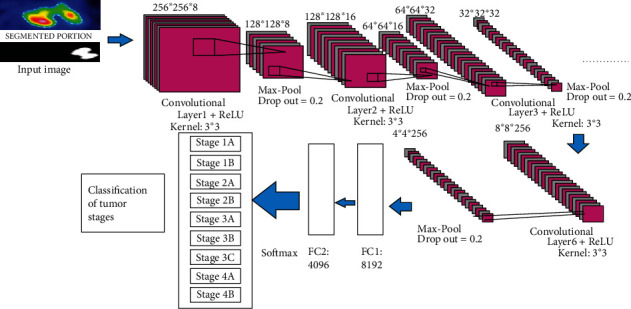
Proposed multilayer CNN architecture.

Convolutional neural network (CNN) is enthused by the human cortex and is one of the supreme prevalent deep learning methods among others. It is considered as a feed-forward network containing multiple layers that implies in one way from input to output. When data is passed through the process layer, useful features are extracted from input data, and the result is shown in output layers. In healthcare, it is used for disease detection from sample tissues. It promotes read structures usually difficult to understand by human medical experts. In the proposed CNN architecture, it results in the classification of lung tumor stages as 9 classes which is described from Figure 10.

The DICOM lung image and PET scan image were given as input to our proposed M-CNN method. Herewith, we have used six CNN layers and each layer consists of 1 convolutional layer and 1 max-pool layer. The proposed M-CNN algorithm helps to classify the lung tumor stages with k3 × 3 kernel function which were examined. We will train our model in each of the 30 epochs. We can reduce our learning rate *μ* in each of the 10 epochs with the factor value of 10. At first, our input image of (height × width × depth) 256 × 256 × 8 pixels is given to our M-CNN with dropout 0.2 and 4096 units of fully connected layer used for classification and leads to 97% accuracy in the lung tumor stage classification. Therefore, our proposed method helps to reduce the error function *E*(*ø*) and for accurate lung tumor stage prediction. The “Softmax and ReLU” function acts as activation function which is described as

(7) $$\begin{array}{c} Z=\mathrm{Max}\left(0,y\right). \end{array}$$

The error function is expressed as

(8) $$\begin{array}{c} E\left(\text{ø}\right)=-\overset{m}{\underset{C=1}{\sum }}Z_{i,c\;\log\left(n_{i,c}\right)}. \end{array}$$

Hence, *Z*_*i*,*c*_ is a binary indication like 0 or 1, *n*_*i*,*c*_ indicates probability prediction like 0 or 1, and *m* refers to number of class.

In terms of the number of convolutional layers used in this model, our proposed M-CNN method has been widely adopted with various configurations. First, 1 convolution layer was used to test CNN, and the analysis was done. After that, CNN with 2 layers was developed, and their findings were evaluated. The method had been used until the model's output was effective and more accurate. Six convolutional layers with max-pooling made up with ReLU and got a final M-CNN model, which would have been extremely viable based on output results. In the outcome portion, the parametric results of every model increment have also been provided with the more accurate result in lung tumor stage classification.

In terms of stage classifications, lung tumor is distinct among other tumor types. The majority of lung tumor stage classification methods are mostly based on experience and consensus. The International Association for the Study of Lung Cancer (IASLC) created a lung tumor stage classification system based on detailed statistical methods of a global database of more than 100,000 cases. This research was presented with TU, LN, and DM components, as well as the stage categories (Tables 5 and 6), methodology, and testing for non–small-cell lung cancer and small cell lung cancer. In Table 7, classification results of performance metrics such as sensitivity, specificity, and accuracy were given by the M-CNN technique with ACM and without the ACM model. These results show that the stage classification with the ACM model achieves more accuracy than without ACM. In this work, we used 9 stages of classification of lung tumor with respect to size and location. If we send the CT/PET scan images to the M-CNN model, we got the less accuracy in stage classification. Therefore, we used the ACM model for tumor portion segmentation and sent that segmented image to our M-CNN model to achieve more accuracy.

### 3.5. LC-Cloud Framework

**Figure 11:**
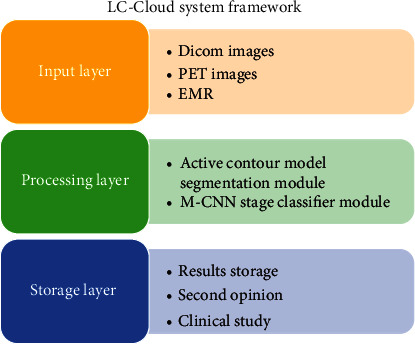
LC-Cloud conceptual architecture.

This section described about the proposed new LC-Cloud system as a lower-level server based on “Saas” used for analysis and storing the patient's diagnosis records for study purpose and for virtual diagnosis purpose (i.e., E-treatment). The main motivation of this LC-Cloud is (i) to improve the diagnosis performance of E-treatment in regional medical care system, (ii) to make use of this patient's records for second opinion or clinical study purpose, and (iii) to make fast processing in real time with low cost. Generally, cancer patients are diagnosed through multiple physicians from various sectors such as neurology, surgeons, and cancer specialist with the help of their personal records which have been stored in their local servers. Conventionally, their record is comprised of numerous images (either DICOM or PET or thermal imaging formats) which are massive computational intensive and demand more memory for storage purpose. In the current scenario, many local workstations are used for such analysis and storing the medical records which greatly rises the cost for installation, maintenance, and reconfiguration process. To address these complexities, researchers are initiated to adopt the cloud computing services due to its ability of fast processing, high-level storage, flexibility in handling the data, and also easily accessible through the internet worldwide. This significance of cloud systems has been adopted in the medical care industry. Liu et al. developed a new cloud server called “iMage” for particularly processing the medical data [30]. The proposed cloud has three distinct layers for analysis, processing, and communication purpose in order to provide the software services. The limitation of this cloud system is mainly focused on security issues in transferring the medical data. Similarly, Ojog et al. proposed the “m3Dicom” server which is dedicated for handling the DICOM depictions [31]. The limitation of this cloud system is focused only on dental segmentation process. Parsonson et al. also developed a dedicated cloud server called “bCloudronics3D” model which stored the volume rendering data [32]. These existing servers are focused on either analysis or storage purpose of medical data but not mentioned for lung cancer detection process. Motivated by these models, we developed a dedicated LC-Cloud framework for lung tumor analysis, processing, and storage purpose as a lower-level public cloud server. The proposed LC-Cloud system is developed with multiple nodes and communication layer for transferring the data through the internet. The LC-Cloud server architecture is comprised of three layers called “input, processing, and storage layer” which is connected with the firewall and LC-server.  Figure 11 illustrates the conceptual architecture of the proposed LC-Cloud system.

The input layer is designed to adopt any category of lung tumor data such as “DICOM images or PET images or EMRs” directly accessible through users or radiologists through the internet. These data are feed-forwarded into the next layer for processing under certain predefined conditions. The new user data is checked with the LC-Cloud engine for availability match. The conditions are (i) if the new user record is matched with existing file, then it is directly forwarded to physicians and (ii) if the data is not matched, then it is sent to the processing layer for diagnosis and sent to the physician. Finally, the physician results and patient records are gathered in the storage layer for clinical study and for virtual treatment process. The processing layer consists of intellectual algorithms called “ACM segmentation algorithm and M-CNN classifier” for diagnosis purpose.

#### 3.5.1. LC-Cloud Significance

- Useful for patients in terms of fast processing particularly in emergency situation
- The records are analyzed worldwide due to its distributed sharing
- Beneficial for both physicians and patients in terms of cost
- Avoiding the data collection and transferring repetition process which saves the analysis time and improves the performance
- Easily accessible
- Lower-level structure with limited maintenance cost
- Used for clinical study purpose or for future references

**Figure 12:**
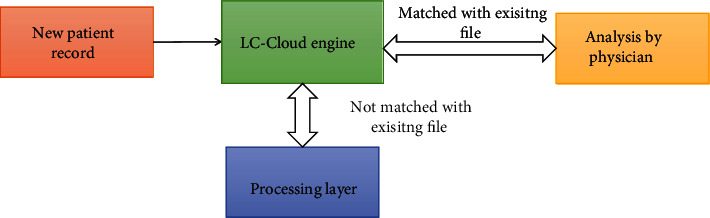
Virtual monitoring and E-treatment by physicians with the LC-Cloud system for new patient.

The LC-Cloud system is beneficial for both physicians and patients in real time. For example, with the use of 4G and 5G connectivity; patients can take precautions from the doctor without visiting and it provides real-time data to the doctor for best treatment. By availing sources like mobile networks, data can be retrieved and sent over devices remotely to physician, radiologists, and external specialists concurrently.  Figure 12 illustrates the working structure of the proposed model for new or unknown lung tumor patient record for analysis.

*LC-Cloud specifications*: the proposed cloud is comprised of three clusters “input cluster, processing cluster, and storage cluster” where each cluster includes different numbers of processors. Each layer includes 4-core processor with 64 GB memory, and these nodes are accessed through a main server. The input data is accessed from a standard database and also adopted from Amazon web service cloud and processed through hardware of LC-Cloud engine. The limitation of the proposed cloud system is capable to analyse only 65 users' record concurrently due to its memory limitation which is not much efficient for large hospital structure. The proposed system can be extended with multiple nodes in the future for large hospitality frameworks with high-level cost, and security issues can be focused in the future.

## 4. Experimental Results and Discussion

*Simulation environment*: the proposed Cloud-LTDSC framework is developed in “Python IDE” with deep learning modules, and various performance metrics are analyzed.

*Experimental setup*: LIDC-IDRI and PET scanned images are adopted for pretraining and testing purposes. The initial analysis such as noise reduction and image segmentation is performed in the hardware PC with i7 processor with “NVIDIA JETSON GPU System-on-module with 256-core NVIDIA Pascal™ GPU architecture with 256 NVIDIA CUDA cores” and memory 64 GB 128-bit LPDDR4 Memory 1866 MHz -59.7 GB/s.”

*Cloud service*: the proposed models with two standard databases are created, and stage classification is performed on Amazon web cloud service due to large database and high-level processing speed is required. The AWS cloud has been used in LC-Cloud system that processes and collects the classification reports that are stored permanently for future diagnosis. Each layer includes 4-core processor with 128 GB memory, and these nodes are accessed through a main server. The input data is accessed from a standard database and also adopted from Amazon web service cloud and processed through hardware of LC-Cloud engine.

**Figure 13:**
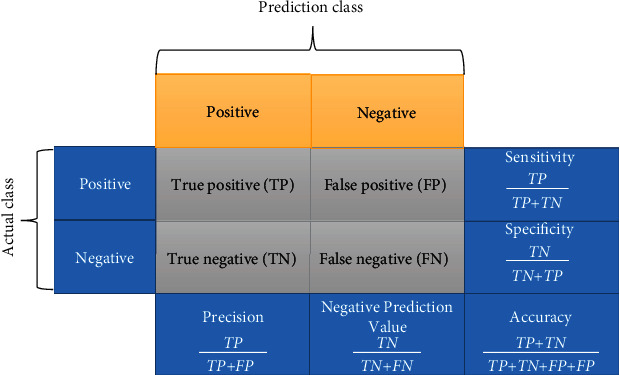
Estimated performance metrics for validating the proposed Cloud-LTDSC model.

**Figure 14:**
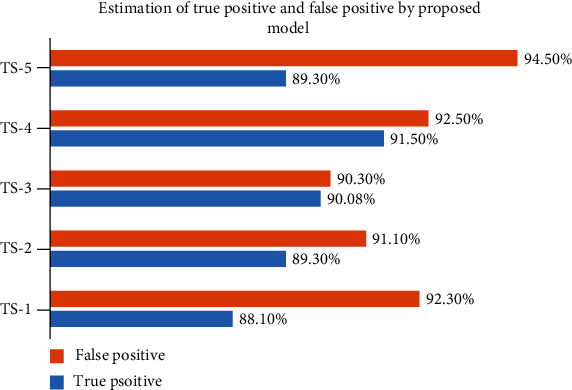
Performance analysis of TP and FP estimated by the proposed model.

**Figure 15:**
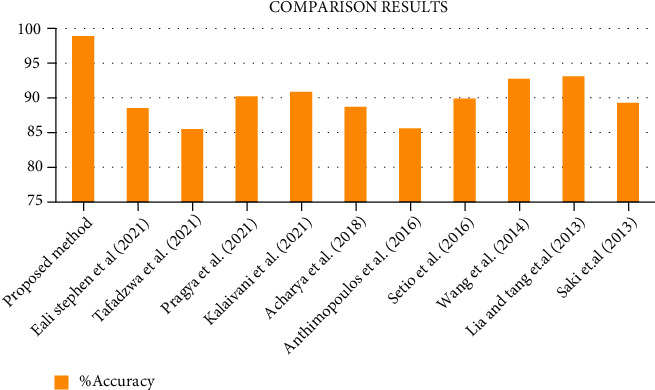
Comparative analysis of the proposed system with existing models.

**Figure 16:**
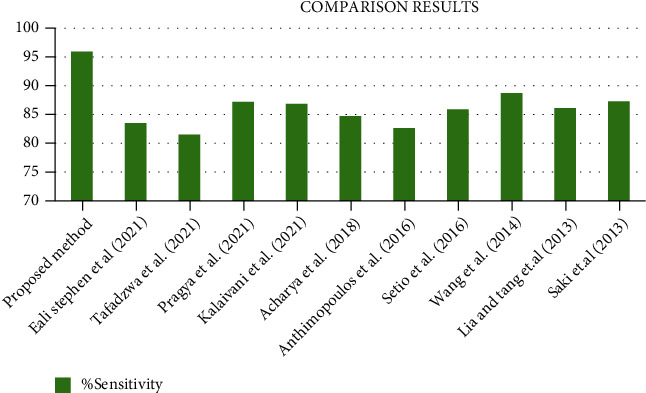
Comparative analysis of specificity metric observed using the proposed model with existing methods.

**Figure 17:**
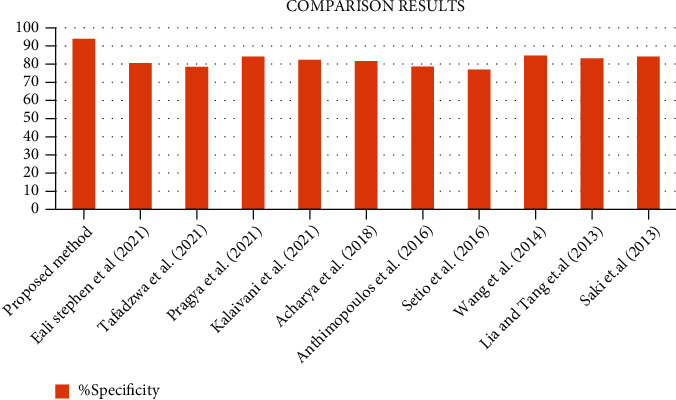
Comparative analysis for cloud with cloud-based system.

From Figure 13, it gives the information about the estimated performance metrics for validating the proposed Cloud-LTDSC model. From Table 8, we compared our performance metrics with the results of an existing system in terms of accuracy, sensitivity, specificity, FPR, and AUC. The performance analysis of testing experiments TS-1, TS-2, TS-3, TS-4, and TS-5 with accuracy (%) of TP, FP, and prediction accuracy from the Figure 14. Each test set is varied with training and testing samples in the range of 20-70% images in training and 10-40% images in testing. The comparative analysis of our proposed model with existing SVM models to deep learning networks is proposed by other authors mentioned above in related works. The proposed model outperformed “10 distinct modules—“AlexNet, CNN, SVM, KNN, RF, 3D-CNN, DenseNet, BPSO-DT, TEP, RNN,” proposed by other authors mentioned in summary table. The metrics are measured for all 11 modules including the M-CNN with ACM segmented model with the performance measures like “accuracy, sensitivity, and specificity” which are shown in Figures 15–17

**Figure 18:**
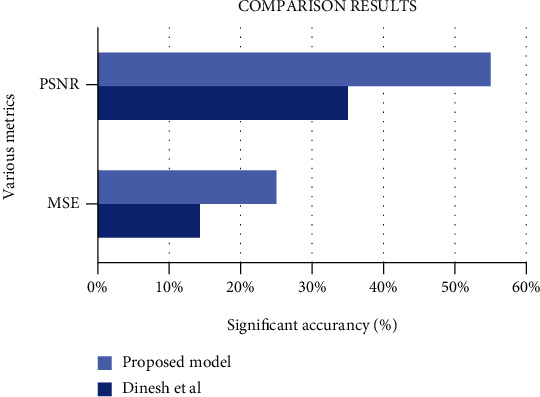
Comparative analysis of the proposed system with existing models.

Figure 18 illustrates the classification accuracy obtained by M-CNN and other deep learning classifiers. The range of accuracy is varied in between 97% and 99.1% for all five test sets with varying training and testing samples. This level of accuracy is achieved in lung tumor stage classification because of exact segmented inputs feed-forwarded into the multilayer network which detected the different classes based on their independent features of each and every segmented image. The test set 2 contained few class 3 images and more class 1 images. Similarly, multistage classification was performed on five distinct test sets which accurately categorized 9 distinct classes.  Figure 15 shows the comparative analysis of sensitivity 95.9% with existing methods. From Figure 16, it shows the comparative analysis of specificity 93.9% with existing methods.

After comparing the performance metrics of our proposed cloud-based healthcare application to existing cloud methods, it can be shown in Figure 17. This happens due to an employment of excellent lung tumor segmentation and also for tumor stage classification approaches, which aid in creating an accurate tumor prediction choice using both traditional lung tumor images and cloud data gathered from remote lung tumor patients. The total elapse time of the proposed method is completed within 2700 seconds, and the time complexity is *O* (*n*^2^), where *n* is the number of pixels in the segmented image.

## 5. Conclusion

In order to lower the lung tumor death rate, this paper focused on constructing a cloud-based lung tumor detector that includes a segmentation module, detection module, and stage classifiers. The suggested Cloud-LTDSC module used unsupervised learning neural networks as a predictor and stage classifier to more precisely identify the tumor. The segmented images are feed-forwarded into the M-CNN model in order to identify its classes and severity level for each patient. The e-record has been generated with patient name, severity level, and tumor details which are transferred through cloud to doctors for virtual monitoring and E-diagnosis automatically. The performance metrics are estimated in terms of accuracy, sensitivity, and specificity and compared with the existing techniques. The results prove that 97% accuracy has been achieved in stage classification for tested segmented images. These deep learning and smart lung tumor models detect the tumor in the earlier stage using the effective virtual monitoring and E-diagnosis. In future work, we will develop and analyse this proposed system for large database and then publish the findings on how the system is capable of safely managing hospital large data and also, this prototype is planned to develop as the complete cloud hardware module in the future work.

## Figures and Tables

**Table 1:** Summary of the proposed model with existing predictors.

Authors	Methodology	Database used	Performance metrics observed accuracy (Acc) and area under the curve (AUC)	Inference
[15]	3D CNN-AlexNet detection algorithm	LUNA	Acc—89%	The drawback of the AlexNet model is tested with 10% of data which is not efficient in medical real-time analysis

[16]	CNN predictor	LUAD	Acc—71%	The limitation of this model is not focused on preprocessing and segmentation which improves the detection accuracy

[9]	SVM, KNN classifiers	LIDC-IDRI, LUNA 16	Acc—91%	Elapse time complexity is high

[17]	DenseNet classifier	LIDC-IDRI	Acc—90.85%	Insignificant database only used

[18]	Unsupervised learning algorithms	LIDC-IDRI	Acc—94.3%	Tissue-based classification model has been focused

[19]	CNN and RNN	LUNA16, ANODE09, and LIDC-IDRI	Acc—91%, AUC—0.78	Modelled as binary classifier and the distinct stages are not focused

[20]	3D-CNN model		Acc—92.65%	These labelled models are detected as benign or malignant

[21]	BPSO-DT	LUNA	Acc—88.25%	Accuracy can be improved with other trending algorithms

[22]	Supervised learning algorithms	LIDC-IDRI	Acc—89.5%	The proposed model focused on cell classification not for segmentation of detection of lung nodules

[23]	Tobacco Exposure Pattern classification model	LUAD	Acc—94.6%	TEP focused on pattern recognition of tobacco as genetic problems

Proposed method	Multistage classifier with cloud connectivity is the new work in the lung tumor detection problem which has been modelled and achieved better results compared to the existing works	Two different databases are used for evaluation LIDC-DICOM and PET scans	Acc—98.9%	The proposed model can be extended in terms of security perspective on the cloud service usages

**Table 2:** Real and simulated nodule characteristics.

Nodule	Diameters (mm)	Contrasts	No. of image	No. of nodules
Real nodules	8–20	24–65	120	112
Simulated nodule	8–12	27–35	634	234
Simulated nodule	4–7	20–23	139	189

**Table 3:** Various types of benign nodules.

Solid nodules	Semisolid nodules	Nonsolid nodules	Total nodules
67	233	24	324

**Table 4:** Performance metrics of accuracy, precision, and recall for 10-fold cross-validation.

*k*-fold	Precision (%)	Recall (%)	Accuracy (%)
1-fold	99.2	97.2	99.6
2-fold	98.8	98.1	99.6
3-fold	97.6	96.5	96.6
4-fold	99.1	97.6	99.8
5-fold	97.5	96.5	96.3
6-fold	97.9	97.7	98.2
7-fold	99.8	96.3	96.2
8-fold	97.7	97.2	99.4
9-fold	97.3	97.6	98.8
10-fold	98.8	98.5	98.2
Mean value	98.37	97.32	98.27

**Table 5:** Lung tumor stages classified based on its size.

Primary tumor (TU)	
TU0	Tumor not occurs
TU1	Tumor size is less than or equal to 3 cm
TU1a	Tumor size is less than or equal to 1 cm
TU1b	Tumor size is more than 1 cm but less than or equal to 2 cm
TU1c	Tumor size is more than 2 cm but less than or equal to 3 cm
TU2	Tumor size is more than 3 cm but less than or equal to 5 cm
TU2a	Tumor size is more than 3 cm but less than or equal to 4 cm
TU2b	Tumor size is more than 4 cm but less than or equal to 5 cm
TU3	Tumor size is more than 5 cm but less than or equal to 7 cm
TU4	Tumor size is more than 7 cm
Region lymph nodes (LN)	
LN_0_	Absence of regional node metastasis
LN_1_	Metastasis is in the ipsilateral peribronchial or perihilar lymph nodes and intrapulmonary nodes
LN_2_	Metastasis is in the ipsilateral mediastinal or subcarinal lymph nodes
LN_3_	Metastasis is in the contralateral mediastinal lymph nodes, perihilar lymph nodes, or supraclavicular nodes
Distant metastasis (DM)	
DM0	Absence of distant metastasis
DM1	Malignant pleural/pericardial effusion or pleural/pericardial nodule or separate tumor nodule in a contralateral lobes
DM2	Distant metastasis was found

**Table 6:** Estimated classification results for test data.

TU/DM	Tumor stages	LN_0_	LN_1_	LN_2_	LN_3_
TU1	TU1a	1A1	2B	3A	3B
	TU1b	1A2	2B	3A	3B
	TU1c	1A3	2B	3A	3B
TU2	TU2a	1B	2B	3A	3B
	TU2b	2A	2B	3A	3B
TU3	TU3	2B	3A	3B	3C
TU4	TU4	3A	3A	3B	3C
DM	DM1	4A	4A	4A	4A
	DM2	4B	4B	4B	4B

**Table 7:** Comparison of performance metrics with and without ACM.

Methods	Sensitivity	Specificity	Accuracy
With ACM	95.9%	93.9%	97.1%
Without ACM	97%	81%	80%

**Table 8:** Comparison of performance metrics with existing methods.

Methods	Medical database	Classifier	%Accuracy	%Sensitivity	%Specificity	False positive rate (FPR)	AUC
Proposed method	LIDC-IDRI/PET	M-CNN	97.1	95.9	93.9	2.1	94
Eali Stephen et al. (2021)	LUNA	3D CNN-AlexNet detection algorithm	89	83.5	80.5	—	91
Tafadzwa et al. (2021)	LUAD	Supervised CNN predictor	90	81.5	78.5	—	71%
Pragya et al. [17]	LIDC-IDRI, LUNA 16	SVM, KNN, and CNN	91	87.2	84.2	—	—
Kalaivani et al. (2021)	LIDC-IDRI	Deep CNN model	90.85	86.85	82.3	—	—
Gopi et al. (2019) [26]	LIDC-IDRI	E-CNN	97	84.7	81.7	1.7/3.8	—
Acharya et al. [33]	EPILEPSIAE	CNN	88.7	82.61	78.61	—	—
Anthimopoulos et al. [34]	ILD CT/HRCT	CNN	85.61	85.9	76.9	—	95%
Setio et al. [35]	LIDC-IDRI	ConvNet	89.9	88.7	84.7	1.0/4.0	
Wang et al. [36]	DDSM	SVM	92.74	86.1	83.1	—	96.50
Liu and Tang et al. [37]	DDSM	SVM	93	87.28	84.2	—	94.39
Saki et al. [38]	MIAS	OWBPE	89.28	95.9	78.5	—	92.80

## Data Availability

The LIDC-IDRI data used in the findings of this study is free publicly available dataset from National Cancer Institute and the Foundation for the National Institute of Health.
